# Third Congress of American Physicians and Surgeons

**Published:** 1893-05

**Authors:** 


THE COMMITTEE OF ARRANGEMENTS,

FRANKLIN H. MARTIN, *Chairman.* S. C. STANTON, *Secretary.*

---

THIRD CONGRESS OF AMERICAN PHYSICIANS AND SURGEONS.—The first meeting of the Executive Committee of the Third Congress of American Physicians and Surgeons was held December 27th, in Philadelphia. The committee was organized by the election of Dr. William Pepper as chairman, and Dr. Newton M. Shaffer as secretary. The following officers of the Congress were elected: Dr. A. L. Loomis, of New York, president; Dr. W. H. Carmalt, of New Haven, secretary; Dr. John Shaw Billings, of Washington, treasurer; and Dr. S. C. Busey, of Washington, chairman of the Committee of Arrangements. It was decided to hold the next meeting in Washington in May, 1894.

---

THE Executive Committee of the American Microscopical Society has decided to hold the next meeting of the society at Madison, Wis., beginning Monday, August 14, 1893.

---

THE American Association for the Advancement of Science has also decided to meet at Madison, Wis., beginning Tuesday, August 15, 1893.

